# Clinical characteristics and epidemiology of intestinal tapeworm infections over the last decade in Tokyo, Japan: A retrospective review

**DOI:** 10.1371/journal.pntd.0006297

**Published:** 2018-02-20

**Authors:** Motoyuki Tsuboi, Kayoko Hayakawa, Hiroshi Yamasaki, Yuichi Katanami, Kei Yamamoto, Satoshi Kutsuna, Nozomi Takeshita, Shuzo Kanagawa, Norio Ohmagari, Yasuyuki Kato

**Affiliations:** 1 Disease Control and Prevention Center, National Center for Global Health and Medicine, Tokyo, Japan; 2 Department of Parasitology, National Institute of Infectious Diseases, Tokyo, Japan; Hospital General, MEXICO

## Abstract

**Background:**

Tapeworm (cestode) infections occur worldwide even in developed countries and globalization has further complicated the epidemiology of such infections. Nonetheless, recent epidemiological data on cestode infections are limited. Our objectives were to elucidate the clinical characteristics and epidemiology of diphyllobothriosis and taeniosis in Tokyo, Japan.

**Methodology/Principal findings:**

We retrospectively reviewed 24 cases of human intestinal cestode infection from January 2006 to December 2015 at a tertiary referral hospital in Tokyo, Japan. The patients included were diagnosed with cestode infection based on morphological and/or molecular identification of expelled proglottids and/or eggs and treated in our hospital. Fifteen and 9 patients were diagnosed with diphyllobothriosis and taeniosis, respectively. The median patient age was 31 years (interquartile range [IQR]: 26–42 years), and 13 (54%) were male. Most of the patients (91.7%) were Japanese. All patients were successfully treated with praziquantel without recurrence. Diphyllobothriosis was caused by *Diphyllobothrium nihonkaiense* in all patients. Taeniosis was due to infection of *Taenia saginata* in 8 [88.9%] patients and *T*. *asiatica* in 1 [11.1%] patient. All patients with taeniosis were infected outside Japan, as opposed to those with diphyllobothriosis, which were domestic. The source locations of taeniosis were mostly in developing regions. The median duration of the stay of the patients with taeniosis at the respective source location was 1 month (IQR: 1–8).

**Conclusions/Significance:**

The cestode infection, especially with *D*. *nihonkaiense*, has frequently occurred, even in Japanese cities, thereby implicating the probable increase in the prevalence of diphyllobothriosis among travelers, as the number of travelers is expected to increase owing to the Tokyo Olympics/Paralympics in 2020. In addition, medical practitioners should be aware of the importance of providing advice to travelers to endemic countries of taeniosis, including the potential risks of infection and preventive methods for these infections.

## Author summary

Tapeworm (cestode) infections occur worldwide even in developed countries. Causative species of cestode infections differ significantly depending on the geographical areas and globalization has further complicated the epidemiology of such infections. Nonetheless, recent epidemiological data on cestode infections are limited. Our objectives were to elucidate the clinical characteristics and epidemiology of diphyllobothriosis and taeniosis in Tokyo, Japan. This study revealed that the cestode infection, especially with *D*. *nihonkaiense*, has frequently occurred, even in Japanese cities, thereby implicating the probable increase in the prevalence of diphyllobothriosis among travelers, as the number of travelers is expected to increase owing to the Tokyo Olympics/Paralympics in 2020. In addition, compared to diphyllobothriosis, taeniosis was occurred mainly among travelers to and from developing countries even for short period of stay (median 1 month [IQR:1–8]). Medical practitioners should be aware of the importance of providing advice to travelers to endemic countries of taeniosis, including the potential risks of infection and preventive methods for these infections.

## Introduction

Tapeworm (cestode) infections, including diphyllobothriosis and taeniosis, occur worldwide even in developed countries. Causative species of cestode infections differ significantly depending on the geographical areas.

Diphyllobothriosis affects an estimated 10–20 million worldwide [1, 2]. Among diphyllobothriosis, *Diphyllobothrium latum* infection due to ingesting undercooked freshwater fish (such as pike, perch, or rainbow trout) is common in Europe, Russia, and South America [3, 4]. However, in Japan, *Diphyllobothrium nihonkaiense* infection associated with consuming raw Pacific salmon is the most prevalent fishborne cestodiasis. Diphyllobothriosis is usually asymptomatic, but anemia due to vitamin B12 deficiency (especially in *D*. *latum* infection), abdominal symptoms (i.e., pain, discomfort, and diarrhea), weight loss, and dizziness have been reported [1–4].

Taeniosis is caused by *Taenia saginata*, *Taenia solium*, and *Taenia asiatica* infections in humans and is associated with ingesting raw or undercooked beef or pork. *T*. *saginata* and *T*. *solium* infections are found worldwide, while *T*. *asiatica* infections are limited to Asia. Even in Japan, autochthonous *T*. *asiatica* infections have been sporadically reported after 2010 [5]. Similar to diphyllobothriosis, although symptoms, such as abdominal cramps and malaise, are described occasionally, symptoms are usually absent.

Globalization has further complicated the epidemiology of cestode infections [6]. Transportation of causative food items, as well as traveling to endemic areas, has caused a rise in cestode infections outside previously known endemic areas [7–9]. For example, in Japan, endemic areas of diphyllobothriosis were previously limited to the coastal region on the Sea of Japan; however, diphyllobothriosis cases have been reported across Japan these days. Nonetheless, recent epidemiological data on cestode infections are limited. The present study aimed to elucidate the clinical characteristics and epidemiology of diphyllobothriosis and taeniosis in Tokyo, Japan.

## Methods

We retrospectively reviewed 24 cases of human intestinal cestode infection from January 2006 to December 2015 at the National Center for Global Health and Medicine (NCGM), a tertiary referral hospital, especially for infectious diseases, in Tokyo, Japan. The patients included were diagnosed with cestode infection based on morphological and/or molecular identification [10, 11] of expelled proglottids and/or eggs and treated in our hospital. Patients’ symptoms, travel history, and history of food consumption were also referred to for diagnosis if necessary. Geographical areas where the patients became infected were determined based on histories of traveling abroad, residential area, and raw fish and meat consumption. Successful treatment was defined as eggs not detected after treatment at the follow-up visit, or confirmed scolex upon treatment with a single dose of praziquantel. Variables associated with epidemiological characteristics were analyzed using the Mann–Whitney’s U test and Fisher’s exact test for continuous and categorical variables, respectively.

### Ethics statement

This study was approved by the Human Research Ethics Committee of NCGM (NCGM-G-001994-00) and all data analyzed were anonymized.

## Results

Fifteen and 9 patients were diagnosed with diphyllobothriosis and taeniosis, respectively. The median patient age was 31 years (interquartile range [IQR]: 26–42 years), and 13 (54%) were male (Table 1). Most of the patients (91.7%) were Japanese. All patients were treated with praziquantel (600 mg once daily, except 1 with *D*. *nihonkaiense* treated with 1500 mg based on decision by an attending physician). No patient experienced recurrence.

**Table 1:** Demographic and clinical characteristics of patients with diphyllobothriosis and taeniosis.

No.	Age	Sex	Residence	Species	Presumed infection site (Length of Stay*^1^)	Sources of infection	Symptoms other than passing proglottids*^2^	EOS (/μL)	Eggs	Molecular diagnosis	Therapy regimen*^3^	Expelled proglottids /scolex after therapy	Recurrence
1	30	F	Japan	*D*. *nihonkaiense*	Japan	Sushi*^4^	Abdominal pain, ABS, diarrhea	135	+	+	Praziquantel 600 mg	+ / +	−
2	23	F				Sushi*^4^	−	189	+	+		+ / +	−
3	42	M				Sashimi	−	NA	+	+		+ / +	−
4	41	M				Sashimi*^4^	−	NA	+	+		+ / −	−
5	33	M				Salmon	−	67	+	+		+ / −	−
6	36	F				Sushi	−	NA	+	+		+ / −	−
7	17	M				Sushi*^4^	−	127	−	+		+ / +	−
8	45	F				Salmon	Abdominal pain	0	−	+		− / −	NA
9	22	M				Salmon,	−	NA	−	+	Praziquantel 1500 mg	− / −	−
10	23	M				Sushi, Sashimi*^4^	ABS, diarrhea	NA	+	−	Praziquantel 600 mg	+ / +	−
11	26	F				Sashimi	−	NA	+	−		+ / +	−
12	28	F				Sushi*^4^	Abdominal pain, diarrhea	NA	+	−		− / −	−
13	30	M				Salmon	−	320	+	−		− / −	−
14	7	M				NA	−	50	+	−		− / −	−
15*^5^	32	M				Salmon	Body weight loss	NA	−	−		− / −	NA
16	26	F		*T*. *saginata*	Indonesia (1 wk) / Laos (7 M)*^6^	Fresh vegetable	−	NA	+	+		+ / +	−
17	42	F			France (2 M)	Raw beef	Diarrhea, abdominal discomfort	280	−	+		+ / −	−
18	52	F			Vietnam (1 M)	Undercooked beef	Perianal discomfort	113	+	+		+ / −	NA
19	51	M			Ethiopia (1 M)	Raw beef	−	NA	−	+		+ / −	NA
20	29	F			Kenya (2 wk)	Raw beef	−	NA	+	−		+ / −	NA
21	32	F			Ethiopia (8 M)	Raw beef	−	152	+	−		+ / −	NA
22	60	M			Jordan (1 M)	Raw beef	−	NA	+	+		− / −	−
23	45	M	Nigeria		UAE (NA)	Raw beef	−	813	+	+		+ / +	−
24	29	M	Philippines	*T*. *asiatica*	Philippines (29 Y)	Raw pork liver	Abdominal pain, diarrhea, itchy rash	356	NA	+		+ / −	NA

*1 Length of stay at presumed infection site was not described among patients with diphyllobothriosis because they reside in Japan.

*2 All patients had passing proglottids.

*3 All therapy regimens were given as single doses, combined with ingestion of sennoside (on the night before) and magnesium citrate (a few hours before and after).

*4 Including salmon

*5 Diagnosed based on the morphology of the parasite, and travel and work history

*6 Presumed infection site was indiscernible

Abbreviations. ABS: Abdominal bloating sensation, EOS: eosinophils, NA: not available, UAE: United Arab Emirates

Diphyllobothriosis was caused by *D*. *nihonkaiense* in all patients. Fourteen (93.3%) patients presented with histories of consuming raw salmon. Prepatent periods could not be estimated in patients with diphyllobothriosis because most of them regularly consumed raw fish. In 9 of 15 (60%) cases, the diagnosis was confirmed through cestode *cox1* gene sequencing. Four (26.7%) patients presented with abdominal symptoms, such as pain/discomfort and diarrhea; one (6.7%) exhibited weight loss. No patient had eosinophilia. After treatment, scoleces were detected in only 6 (40%) patients.

Taeniosis was due to infection of *T. saginata* in 8 (88.9%) patients and *T*. *asiatica* in 1 (11.1%) patient; these patients had consumed raw beef and pork liver, respectively. The median prepatent period of taeniosis was 4 months (IQR: 1.5–5). In 7 of 9 (77.8%) cases, the diagnosis was confirmed via cestode *cox1* gene sequencing. Three (33.3%) patients presented with abdominal symptoms; no patient exhibited weight loss. Eosinophilia was detected in only 1 patient with *T*. *saginata* infection of 5 (20%) patients with taeniosis for whom laboratory test results were available. After treatment, scoleces were detected in only 2 (22.2%) patients.

Patients with taeniosis were slightly older than those with diphyllobothriosis, and tended to display a longer duration between symptom onset and the first clinical visit (Table 2). All patients with taeniosis were infected outside Japan, as opposed to those with diphyllobothriosis, which were domestic. The source locations of taeniosis infection were mostly in developing regions such as Africa, Southeast Asia, or the Middle East. The median duration of the stay of the patients with taeniosis at the respective source location was 1 month (IQR: 1–8).

**Table 2:** Comparison of epidemiological characteristics of diphyllobothriosis and taeniosis.

	Diphyllobothriosis (n = 15)	Taeniosis (n = 9)	P value
Median age (IQR), years	30 (23–36)	42 (29–52)	0.04
Male gender (%)	9 (60)	4 (44.4)	0.68
Prepatent period (month, IQR)	NA	4 (1.5–5)	NA
Median days between onset of symptoms and first visit (IQR)	5 (1–34)	14 (7–150)	0.06
Domestic infection (%)	15 (100)	0 (0)	<0.001
Length of the stay in endemic area (month, IQR)	NA	1 (0.75–5)	NA
Abdominal symptoms (%)	4 (26.7)	3 (33.3)	1.0
Detection of eggs (%)	12 (80)	4 (44.4)	0.18
Expelled scolex after therapy (%)	6 (40)	2 (22.2)	0.66
Recurrence (%)	0 (0)	0 (0)	1.0

Values are no. (%) unless otherwise indicated. Abbreviation. IQR, interquartile range. NA, not available.

## Discussion

This study highlights that the cestode infection, especially with *D*. *nihonkaiense*, has frequently occurred, even in Japanese cities. In Japan, 114 cases infected with *D*. *nihonkaiense* from all over the country were identified at the National Institute of Infectious Diseases (NIID), Tokyo, Japan, and 325 cases were reported on the medical articles between January 2007 and March 2017 [12]. These facts mean that approximately 40 cases of autochthonous *D*. *nihonkaiense* infection were annually reported over the last decade in Japan. However, the actual number is expected to be much higher and it would probaply be several times as many as reported number annually because of the cases diagnosed at the other institute or unreported [12]. As traditional Japanese meals and restaurants have become increasingly popular worldwide and there are no regulation that recommends to freeze fishes before consuming it raw in Japan, like that of the US Food and Drug Administration [13], it would be valuable to advise people not only Japanese consumers and manufacturers but also to travelers from foreign countries about the potential risks of eating raw salmon without freezing. In the present study, diphyllobothriosis was caused by *D*. *nihonkaiense* in all patients, who were presumed to be infected in Japan. These results were consistent with the fact that *D*. *latum* infection, confirmed through molecular analysis, from humans has not been reported in Japan [14].

Determining the geographical area of infection is helpful for identifying the cestode species. The estimated infection sites were significantly different between taeniosis and diphyllobothriosis. In the present study, the finding that all taeniosis infections occurred in foreign, mainly developing, countries reflects the fact that, in Japan, taeniosis has mainly been reported among travelers to or from endemic areas. From January 1990 to March 2017, eighty-eight cases of taeniosis from all over the country were identified at the NIID and 95 cases were published on medical articles. Based on these data, approximately seven cases with taeniosis have been reported annually in recent years in Japan [12]. Along with diphyllobothriosis, the actual number of taeniosis is expected to be much higher because the cases of taeniosis should have been under-reported [12]. In addition, although imported *T*. *solium*, including cysticercosis, and domestic *T*. *asiatica* infections were not identified in this study, they can occur in Japan [5]. For instance, in 2010, nineteen out of twenty patients infected with *T*. *asiatica* were Japanese nationals residing in the Kanto region where Tokyo belongs to, and fifteen patients stated that they frequently ate raw pig liver. In addition, sixteen of them had never been overseas or, if they had undertaken any international travel, they traveled to countries where *T*. *asiatica* is not endemic [5].

This study showed two other important findings. First, the lengths of the stay in endemic areas in taeniosis cases were relatively short. This finding highlights the importance of pre-travel education on appropriate food selections for those planning to stay in endemic countries, even for short periods. Second, patients with taeniosis were significantly older to those with diphyllobothriosis, probably because middle-aged people, rather than younger people, are likely to travel abroad for much longer periods for work purposes. In the present study, 6 out of 9 patients with taeniosis visited endemic areas for work.

*D*. *nihonkaiense* infection often occurs even in cities in Japan, thereby implicating the probable increase in the prevalence of diphyllobothriosis among travelers, as the number of travelers is expected to increase owing to the Tokyo Olympics/Paralympics in 2020. In addition, considering the increasing number of travelers to foreign countries where taeniosis is endemic, medical practitioners should be aware of the importance of providing advice to travelers including the potential risks of infection, and preventive methods for these infections should be considered.

## Supporting information

S1 Checklist STROBE checklist. (DOCX)
